# Supplementary material for: A Highly Selective Turn-On Fluorescent Probe for the Detection of Zinc
Source: Molecules. 2021 Jun 23;26(13):3825. doi: 10.3390/molecules26133825 (PMC8270291; doi:10.3390/molecules26133825)

checkCIF/PLATON report

Structure factors have been supplied for datablock(s) L-Cu

THIS REPORT IS FOR GUIDANCE ONLY. IF USED AS PART OF A REVIEW PROCEDURE FOR PUBLICATION, IT SHOULD NOT REPLACE THE EXPERTISE OF AN EXPERIENCED CRYSTALLOGRAPHIC REFEREE.

No syntax errors found.   CIF dictionary   Interpreting this report

Datablock: L-Cu

```
Bond precision:  C-C = 0.0055 A          Wavelength=0.71073

Cell:             a=7.6261(8)     b=12.8842(15)      c=19.144(2)
                  alpha=90        beta=90            gamma=90
Temperature:      302 K

                Calculated                 Reported
Volume          1881.0(4)                  1881.1(4)
Space group     P 21 21 21                 P 21 21 21
Hall group      P 2ac 2ab                  P 2ac 2ab
Moiety formula  C19 H24 Cu N2 O3, H2 O     C19 H24 Cu N2 O3, H2 O
Sum formula     C19 H26 Cu N2 O4           C19 H24 Cu N3 O3
Mr              409.97                     405.95
Dx,g cm-3       1.448                      1.433
Z               4                          4
Mu (mm-1)       1.187                      1.184
F000            860.0                      848.0
F000'           861.59
h,k,lmax        10,17,25                   10,17,25
Nref            4697[ 2679]                4634
Tmin,Tmax       0.762,0.808                0.489,0.746
Tmin'           0.762

Correction method= # Reported T Limits: Tmin=0.489 Tmax=0.746
AbsCorr = MULTI-SCAN

Data completeness= 1.73/0.99      Theta(max)= 28.348

R(reflections)= 0.0382( 3547)     wR2(reflections)= 0.0761( 4634)

S = 1.029                  Npar= 241
```

The following ALERTS were generated. Each ALERT has the format
test-name_ALERT_alert-type_alert-level.
Click on the hyperlinks for more details of the test.

Alert level B

```
PLAT035_ALERT_1_B _chemical_absolute_configuration Info  Not Given     Please Do !
```

Alert level C

```
PLAT041_ALERT_1_C Calc. and Reported SumFormula    Strings  Differ     Please Check
PLAT043_ALERT_1_C Calculated and Reported Mol. Weight Differ by ..       4.02 Check
PLAT068_ALERT_1_C Reported F000 Differs from Calcd (or Missing)...     Please Check
PLAT911_ALERT_3_C Missing FCF Refl Between Thmin & STh/L=    0.600          2 Report
```

Alert level G

```
FORMU01_ALERT_1_G  There is a discrepancy between the atom counts in the
            _chemical_formula_sum and _chemical_formula_moiety. This is
            usually due to the moiety formula being in the wrong format.
            Atom count from _chemical_formula_sum:   C19 H24 Cu1 N3 O3
            Atom count from _chemical_formula_moiety:C19 H26 Cu1 N2 O4
FORMU01_ALERT_2_G  There is a discrepancy between the atom counts in the
            _chemical_formula_sum and the formula from the _atom_site* data.
            Atom count from _chemical_formula_sum:C19 H24 Cu1 N3 O3
            Atom count from the _atom_site data:  C19 H26 Cu1 N2 O4
CELLZ01_ALERT_1_G Difference between formula and atom_site contents detected.
CELLZ01_ALERT_1_G ALERT: Large difference may be due to a
            symmetry error - see SYMMG tests
           From the CIF: _cell_formula_units_Z    4
           From the CIF: _chemical_formula_sum  C19 H24 Cu N3 O3
           TEST: Compare cell contents of formula and atom_site data

           atom    Z*formula  cif sites diff
           C         76.00     76.00    0.00
           H         96.00    104.00   -8.00
           Cu         4.00      4.00    0.00
           N         12.00      8.00    4.00
           O         12.00     16.00   -4.00
PLAT007_ALERT_5_G Number of Unrefined Donor-H Atoms ..............          2 Report
PLAT720_ALERT_4_G Number of Unusual/Non-Standard Labels ..........          2 Note
PLAT791_ALERT_4_G Model has Chirality at C9          (Sohnke SpGr)          R Verify
PLAT791_ALERT_4_G Model has Chirality at C14         (Sohnke SpGr)          R Verify
PLAT883_ALERT_1_G No Info/Value for _atom_sites_solution_primary .     Please Do !
PLAT910_ALERT_3_G Missing # of FCF Reflection(s) Below Theta(Min).          3 Note
PLAT912_ALERT_4_G Missing # of FCF Reflections Above STh/L=  0.600          7 Note
PLAT913_ALERT_3_G Missing # of Very Strong Reflections in FCF ....          1 Note
PLAT941_ALERT_3_G Average HKL Measurement Multiplicity ...........        3.4 Low
PLAT978_ALERT_2_G Number C-C Bonds with Positive Residual Density.          0 Info
PLAT992_ALERT_5_G Repd & Actual _reflns_number_gt Values Differ by          2 Check
```

```
   0 ALERT level A = Most likely a serious problem - resolve or explain
   1 ALERT level B = A potentially serious problem, consider carefully
   4 ALERT level C = Check. Ensure it is not caused by an omission or oversight
  15 ALERT level G = General information/check it is not something unexpected

   8 ALERT type 1 CIF construction/syntax error, inconsistent or missing data
   2 ALERT type 2 Indicator that the structure model may be wrong or deficient
   4 ALERT type 3 Indicator that the structure quality may be low
   4 ALERT type 4 Improvement, methodology, query or suggestion
   2 ALERT type 5 Informative message, check
```

It is advisable to attempt to resolve as many as possible of the alerts in all categories. Often the minor alerts point to easily fixed oversights, errors and omissions in your CIF or refinement strategy, so attention to these fine details can be worthwhile. In order to resolve some of the more serious problems it may be necessary to carry out additional measurements or structure refinements. However, the purpose of your study may justify the reported deviations and the more serious of these should normally be commented upon in the discussion or experimental section of a paper or in the "special_details" fields of the CIF. checkCIF was carefully designed to identify outliers and unusual parameters, but every test has its limitations and alerts that are not important in a particular case may appear. Conversely, the absence of alerts does not guarantee there are no aspects of the results needing attention. It is up to the individual to critically assess their own results and, if necessary, seek expert advice.

Publication of your CIF in IUCr journals

A basic structural check has been run on your CIF. These basic checks will be run on all CIFs submitted for publication in IUCr journals (*Acta Crystallographica*, *Journal of Applied Crystallography*, *Journal of Synchrotron Radiation*); however, if you intend to submit to *Acta Crystallographica Section C* or *E* or *IUCrData*, you should make sure that full publication checks are run on the final version of your CIF prior to submission.

Publication of your CIF in other journals

Please refer to the *Notes for Authors* of the relevant journal for any special instructions relating to CIF submission.

PLATON version of 05/12/2020; check.def file version of 05/12/2020

Datablock L-Cu - ellipsoid plot

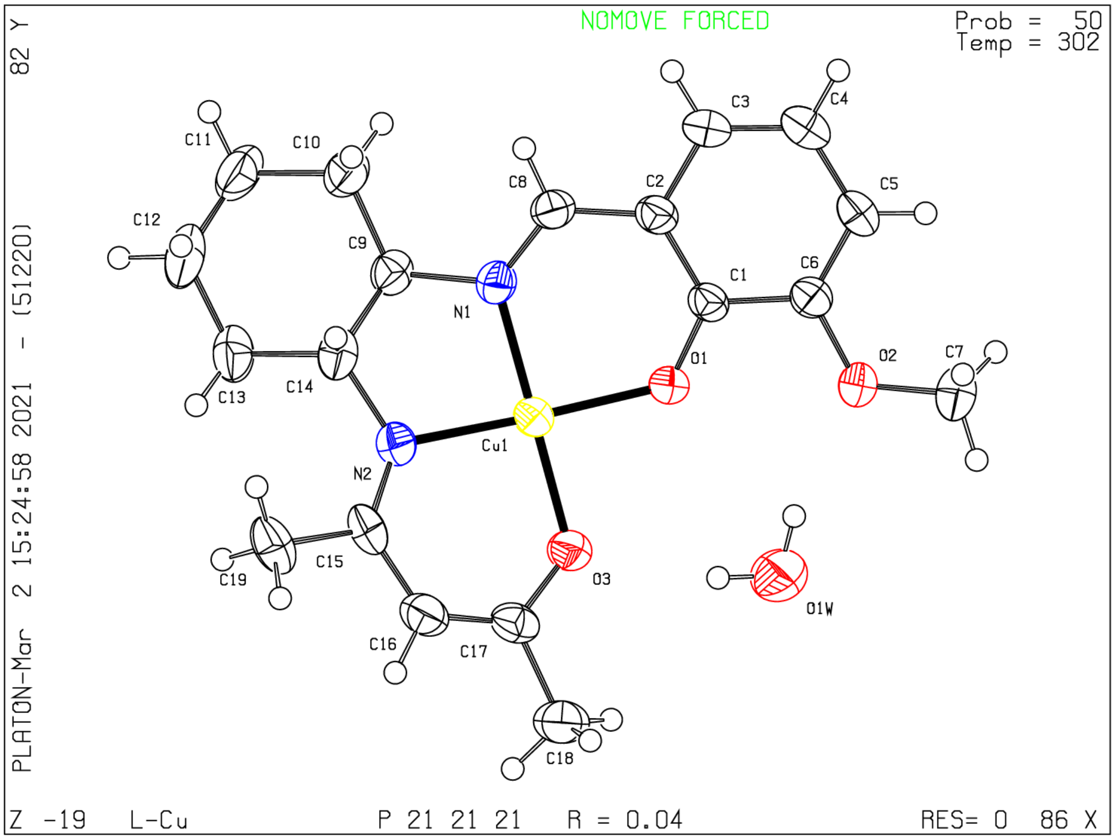

Supplement: Supplementary file 1 [file molecules-26-03825-s001.zip › Crystal/checkcif-Cu.pdf]
