# Supplementary material for: A Highly Selective Turn-On Fluorescent Probe for the Detection of Zinc
Source: Molecules. 2021 Jun 23;26(13):3825. doi: 10.3390/molecules26133825 (PMC8270291; doi:10.3390/molecules26133825)

checkCIF/PLATON report

Structure factors have been supplied for datablock(s) 1_L

THIS REPORT IS FOR GUIDANCE ONLY. IF USED AS PART OF A REVIEW PROCEDURE FOR PUBLICATION, IT SHOULD NOT REPLACE THE EXPERTISE OF AN EXPERIENCED CRYSTALLOGRAPHIC REFEREE.

No syntax errors found. CIF dictionary Interpreting this report

Datablock: 1_L

```
Bond precision:  C-C = 0.0148 A          Wavelength=0.71073

Cell:              a=17.989(2)      b=17.989(2)      c=12.862(2)
                   alpha=90         beta=90          gamma=90
Temperature:       293 K
```

	Calculated	Reported
Volume	4162.2(11)	4162.2(11)
Space group	P 42 21 2	P 42 21 2
Hall group	P 4n 2n	P 4n 2n
Moiety formula	2(C19 H27 N2 O3), O [+ solvent]	C19 H27 N2 O3, 0.5(O)
Sum formula	C38 H54 N4 O7 [+ solvent]	C19 H27 N2 O3.50
Mr	678.85	339.42
Dx,g cm-3	1.083	1.083
Z	4	8
Mu (mm-1)	0.075	0.075
F000	1464.0	1464.0
F000’	1464.66	
h,k,lmax	21,21,15	21,21,14
Nref	3675[2138]	3594
Tmin,Tmax	0.987,0.991	0.607,0.746
Tmin’	0.983	

```
Correction method= # Reported T Limits: Tmin=0.607 Tmax=0.746
AbsCorr = MULTI-SCAN

Data completeness= 1.68/0.98      Theta(max)= 24.992

R(reflections)= 0.1018( 1749)     wR2(reflections)= 0.3231( 3594)

S = 1.024                   Npar= 226
```

The following ALERTS were generated. Each ALERT has the format
test-name_ALERT_alert-type_alert-level.
Click on the hyperlinks for more details of the test.

Alert level B

```
DIFMN02_ALERT_2_B  The minimum difference density is < -0.1*ZMAX*1.00
            _refine_diff_density_min given =     -0.818
            Test value =     -0.800
RINTA01_ALERT_3_B  The value of Rint is greater than 0.18
            Rint given   0.192
PLAT020_ALERT_3_B The Value of Rint is Greater Than 0.12 .........      0.192 Report
PLAT035_ALERT_1_B _chemical_absolute_configuration Info  Not Given     Please Do !
PLAT098_ALERT_2_B Large Reported Min.  (Negative) Residual Density      -0.82 eA-3
PLAT306_ALERT_2_B Isolated Oxygen Atom (H-atoms Missing ?) .......         O4 Check
PLAT340_ALERT_3_B Low Bond Precision on  C-C Bonds ...............    0.01482 Ang.
PLAT420_ALERT_2_B D-H Without Acceptor       O2       --H2A      .     Please Check
```

Alert level C

```
ABSTY02_ALERT_1_C  An _exptl_absorpt_correction_type has been given without
            a literature citation. This should be contained in the
            _exptl_absorpt_process_details field.
            Absorption correction given as multi-scan
DIFMN03_ALERT_1_C  The minimum difference density is < -0.1*ZMAX*0.75
            The relevant atom site should be identified.
STRVA01_ALERT_4_C           Flack parameter is too small
           From the CIF: _refine_ls_abs_structure_Flack   -2.000
           From the CIF: _refine_ls_abs_structure_Flack_su    6.000
PLAT026_ALERT_3_C Ratio Observed / Unique Reflections (too) Low ..        49% Check
PLAT029_ALERT_3_C _diffrn_measured_fraction_theta_full value Low .      0.974 Why?
PLAT084_ALERT_3_C High wR2 Value (i.e. > 0.25) ..................       0.32 Report
PLAT230_ALERT_2_C Hirshfeld Test Diff for    O2       --C9       .        5.3 s.u.
PLAT234_ALERT_4_C Large Hirshfeld Difference C8       --C9       .       0.16 Ang.
PLAT242_ALERT_2_C Low    'MainMol' Ueq as Compared to Neighbors of         C4 Check
PLAT260_ALERT_2_C Large Average Ueq of Residue Including        O4      0.160 Check
PLAT416_ALERT_2_C Short Intra D-H..H-D       H2       ..H2A      .       1.94 Ang.
                                                      x,y,z  =      1_555 Check
PLAT911_ALERT_3_C Missing FCF Refl Between Thmin & STh/L=    0.594         42 Report
PLAT918_ALERT_3_C Reflection(s) with I(obs) much Smaller I(calc) .          2 Check
```

Alert level G

```
PLAT002_ALERT_2_G Number of Distance or Angle Restraints on AtSite          2 Note
PLAT007_ALERT_5_G Number of Unrefined Donor-H Atoms ..............          3 Report
PLAT032_ALERT_4_G Std. Uncertainty on Flack Parameter Value High .      6.000 Report
PLAT042_ALERT_1_G Calc. and Reported MoietyFormula Strings  Differ     Please Check
PLAT045_ALERT_1_G Calculated and Reported Z Differ by a Factor ...       0.50 Check
PLAT072_ALERT_2_G SHELXL First  Parameter in WGHT  Unusually Large       0.20 Report
PLAT172_ALERT_4_G The CIF-Embedded .res File Contains DFIX Records          1 Report
PLAT199_ALERT_1_G Reported _cell_measurement_temperature ..... (K)        293 Check
PLAT200_ALERT_1_G Reported   _diffrn_ambient_temperature ..... (K)        293 Check
PLAT304_ALERT_4_G Non-Integer Number of Atoms in ..... (Resd  2  )       0.50 Check
PLAT605_ALERT_4_G Largest Solvent Accessible VOID in the Structure        253 A**3
PLAT791_ALERT_4_G Model has Chirality at C5         (Sohnke SpGr)           R Verify
PLAT791_ALERT_4_G Model has Chirality at C6         (Sohnke SpGr)           R Verify
PLAT860_ALERT_3_G Number of Least-Squares Restraints .............          1 Note
PLAT869_ALERT_4_G ALERTS Related to the Use of SQUEEZE Suppressed           ! Info
PLAT870_ALERT_4_G ALERTS Related to Twinning Effects Suppressed ..          ! Info
PLAT883_ALERT_1_G No Info/Value for _atom_sites_solution_primary .     Please Do !
PLAT910_ALERT_3_G Missing # of FCF Reflection(s) Below Theta(Min).          3 Note
```

```
PLAT913_ALERT_3_G Missing # of Very Strong Reflections in FCF ....          1 Note
PLAT916_ALERT_2_G Hooft y and Flack x Parameter Values Differ by .       0.50 Check
PLAT961_ALERT_5_G Dataset Contains no Negative Intensities .......     Please Check
```

```
 0 ALERT level A = Most likely a serious problem - resolve or explain
 8 ALERT level B = A potentially serious problem, consider carefully
13 ALERT level C = Check. Ensure it is not caused by an omission or oversight
21 ALERT level G = General information/check it is not something unexpected

 8 ALERT type 1 CIF construction/syntax error, inconsistent or missing data
11 ALERT type 2 Indicator that the structure model may be wrong or deficient
11 ALERT type 3 Indicator that the structure quality may be low
10 ALERT type 4 Improvement, methodology, query or suggestion
 2 ALERT type 5 Informative message, check
```

It is advisable to attempt to resolve as many as possible of the alerts in all categories. Often the minor alerts point to easily fixed oversights, errors and omissions in your CIF or refinement strategy, so attention to these fine details can be worthwhile. In order to resolve some of the more serious problems it may be necessary to carry out additional measurements or structure refinements. However, the purpose of your study may justify the reported deviations and the more serious of these should normally be commented upon in the discussion or experimental section of a paper or in the "special_details" fields of the CIF. checkCIF was carefully designed to identify outliers and unusual parameters, but every test has its limitations and alerts that are not important in a particular case may appear. Conversely, the absence of alerts does not guarantee there are no aspects of the results needing attention. It is up to the individual to critically assess their own results and, if necessary, seek expert advice.

Publication of your CIF in IUCr journals

A basic structural check has been run on your CIF. These basic checks will be run on all CIFs submitted for publication in IUCr journals (*Acta Crystallographica*, *Journal of Applied Crystallography*, *Journal of Synchrotron Radiation*); however, if you intend to submit to *Acta Crystallographica Section C* or *E* or *IUCrData*, you should make sure that full publication checks are run on the final version of your CIF prior to submission.

Publication of your CIF in other journals

Please refer to the *Notes for Authors* of the relevant journal for any special instructions relating to CIF submission.

PLATON version of 05/12/2020; check.def file version of 05/12/2020

Datablock 1_L - ellipsoid plot

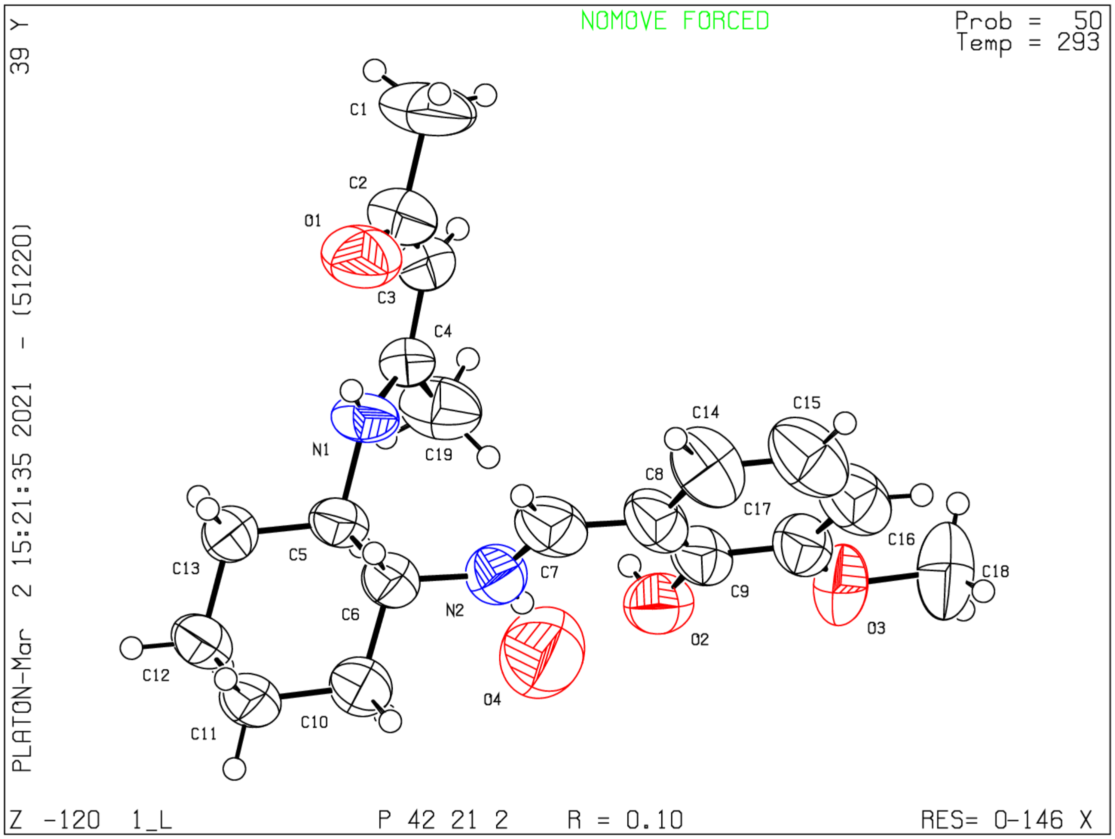

Supplement: Supplementary file 1 [file molecules-26-03825-s001.zip › Crystal/checkcif-L.pdf]
