# Supplementary material for: A Highly Selective Turn-On Fluorescent Probe for the Detection of Zinc
Source: Molecules. 2021 Jun 23;26(13):3825. doi: 10.3390/molecules26133825 (PMC8270291; doi:10.3390/molecules26133825)

checkCIF/PLATON report

Structure factors have been supplied for datablock(s) Cu-Zn

THIS REPORT IS FOR GUIDANCE ONLY. IF USED AS PART OF A REVIEW PROCEDURE FOR PUBLICATION, IT SHOULD NOT REPLACE THE EXPERTISE OF AN EXPERIENCED CRYSTALLOGRAPHIC REFEREE.

No syntax errors found. CIF dictionary Interpreting this report

Datablock: Cu-Zn

```
Bond precision:  C-C = 0.0148 A          Wavelength=0.71073

Cell:           a=9.9812(11)    b=19.966(2)         c=11.0609(12)
                alpha=90        beta=105.840(4)     gamma=90
Temperature:    293 K

                Calculated                 Reported
Volume          2120.6(4)                  2120.6(4)
Space group     P 21                       P 1 21 1
Hall group      P 2yb                      P 2yb
Moiety formula  C38 H50 Cl4 Cu2 N4 O6 Zn2  2(C19 H25 Cl2 Cu N2 O3 Zn)
Sum formula     C38 H50 Cl4 Cu2 N4 O6 Zn2  C38 H50 Cl4 Cu2 N4 O6 Zn2
Mr              1058.50                    1058.44
Dx,g cm-3       1.658                      1.658
Z               2                          2
Mu (mm-1)       2.408                      2.408
F000            1080.0                     1080.0
F000'           1083.87
h,k,lmax        11,23,13                   11,23,13
Nref            7447[ 3840]                7370
Tmin,Tmax       0.567,0.618                0.486,0.645
Tmin'           0.426

Correction method= # Reported T Limits: Tmin=0.486 Tmax=0.645
AbsCorr = MULTI-SCAN

Data completeness= 1.92/0.99      Theta(max)= 24.996

R(reflections)= 0.0718( 6072)     wR2(reflections)= 0.1799( 7370)

S = 0.999                  Npar= 511
```

The following ALERTS were generated. Each ALERT has the format
test-name_ALERT_alert-type_alert-level.
Click on the hyperlinks for more details of the test.

Alert level B

```
PLAT035_ALERT_1_B _chemical_absolute_configuration Info  Not Given     Please Do !
PLAT936_ALERT_2_B The Embedded .res File Includes a DAMP Command .      600.0 Report
```

Alert level C

```
ABSTY02_ALERT_1_C  An _exptl_absorpt_correction_type has been given without
            a literature citation. This should be contained in the
            _exptl_absorpt_process_details field.
            Absorption correction given as multi-scan
RINTA01_ALERT_3_C  The value of Rint is greater than 0.12
            Rint given   0.177
PLAT020_ALERT_3_C The Value of Rint is Greater Than 0.12 .........      0.177 Report
PLAT090_ALERT_3_C Poor Data / Parameter Ratio (Zmax > 18) ........       7.50 Note
PLAT241_ALERT_2_C High   'MainMol' Ueq as Compared to Neighbors of        Cl2 Check
PLAT241_ALERT_2_C High   'MainMol' Ueq as Compared to Neighbors of        Cl3 Check
PLAT241_ALERT_2_C High   'MainMol' Ueq as Compared to Neighbors of         C7 Check
PLAT341_ALERT_3_C Low Bond Precision on  C-C Bonds ...............    0.01479 Ang.
PLAT362_ALERT_2_C Short  C(sp3)-C(sp2) Bond  C2       - C3       .       1.37 Ang.
PLAT362_ALERT_2_C Short  C(sp3)-C(sp2) Bond  C21      - C22      .       1.37 Ang.
PLAT790_ALERT_4_C Centre of Gravity not Within Unit Cell: Resd.  #          1 Note
              C38 H50 Cl4 Cu2 N4 O6 Zn2
PLAT911_ALERT_3_C Missing FCF Refl Between Thmin & STh/L=    0.595          8 Report
PLAT977_ALERT_2_C Check Negative Difference Density on H22B             -0.35 eA-3
```

Alert level G

```
PLAT042_ALERT_1_G Calc. and Reported MoietyFormula Strings  Differ     Please Check
PLAT072_ALERT_2_G SHELXL First  Parameter in WGHT  Unusually Large       0.10 Report
PLAT115_ALERT_5_G ADDSYM Detects Noncrystallographic Inversion ...        85% Check
PLAT199_ALERT_1_G Reported _cell_measurement_temperature ..... (K)        293 Check
PLAT200_ALERT_1_G Reported   _diffrn_ambient_temperature ..... (K)        293 Check
PLAT232_ALERT_2_G Hirshfeld Test Diff (M-X)  Cu2      --Cl2      .        6.6 s.u.
PLAT343_ALERT_2_G Unusual sp3      Angle Range in Main Residue for         C3 Check
PLAT343_ALERT_2_G Unusual sp3      Angle Range in Main Residue for        C22 Check
PLAT791_ALERT_4_G Model has Chirality at C6          (Sohnke SpGr)          R Verify
PLAT791_ALERT_4_G Model has Chirality at C11         (Sohnke SpGr)          R Verify
PLAT791_ALERT_4_G Model has Chirality at C25         (Sohnke SpGr)          R Verify
PLAT791_ALERT_4_G Model has Chirality at C30         (Sohnke SpGr)          R Verify
PLAT794_ALERT_5_G Tentative Bond Valency for Zn1       (II)      .       2.02 Info
PLAT794_ALERT_5_G Tentative Bond Valency for Zn2       (II)      .       2.04 Info
PLAT794_ALERT_5_G Tentative Bond Valency for Cu1       (II)      .       2.35 Info
PLAT794_ALERT_5_G Tentative Bond Valency for Cu2       (II)      .       2.37 Info
PLAT883_ALERT_1_G No Info/Value for _atom_sites_solution_primary .     Please Do !
PLAT909_ALERT_3_G Percentage of I>2sig(I) Data at Theta(Max) Still        52% Note
PLAT961_ALERT_5_G Dataset Contains no Negative Intensities .......     Please Check
PLAT978_ALERT_2_G Number C-C Bonds with Positive Residual Density.          0 Info
```

0 **ALERT level A** = Most likely a serious problem - resolve or explain
2 **ALERT level B** = A potentially serious problem, consider carefully
13 **ALERT level C** = Check. Ensure it is not caused by an omission or oversight
20 **ALERT level G** = General information/check it is not something unexpected

6 ALERT type 1 CIF construction/syntax error, inconsistent or missing data
12 ALERT type 2 Indicator that the structure model may be wrong or deficient
6 ALERT type 3 Indicator that the structure quality may be low
5 ALERT type 4 Improvement, methodology, query or suggestion
6 ALERT type 5 Informative message, check

It is advisable to attempt to resolve as many as possible of the alerts in all categories. Often the minor alerts point to easily fixed oversights, errors and omissions in your CIF or refinement strategy, so attention to these fine details can be worthwhile. In order to resolve some of the more serious problems it may be necessary to carry out additional measurements or structure refinements. However, the purpose of your study may justify the reported deviations and the more serious of these should normally be commented upon in the discussion or experimental section of a paper or in the "special_details" fields of the CIF. checkCIF was carefully designed to identify outliers and unusual parameters, but every test has its limitations and alerts that are not important in a particular case may appear. Conversely, the absence of alerts does not guarantee there are no aspects of the results needing attention. It is up to the individual to critically assess their own results and, if necessary, seek expert advice.

Publication of your CIF in IUCr journals

A basic structural check has been run on your CIF. These basic checks will be run on all CIFs submitted for publication in IUCr journals (*Acta Crystallographica*, *Journal of Applied Crystallography*, *Journal of Synchrotron Radiation*); however, if you intend to submit to *Acta Crystallographica Section C* or *E* or *IUCrData*, you should make sure that full publication checks are run on the final version of your CIF prior to submission.

Publication of your CIF in other journals

Please refer to the *Notes for Authors* of the relevant journal for any special instructions relating to CIF submission.

PLATON version of 05/12/2020; check.def file version of 05/12/2020

Datablock Cu-Zn - ellipsoid plot

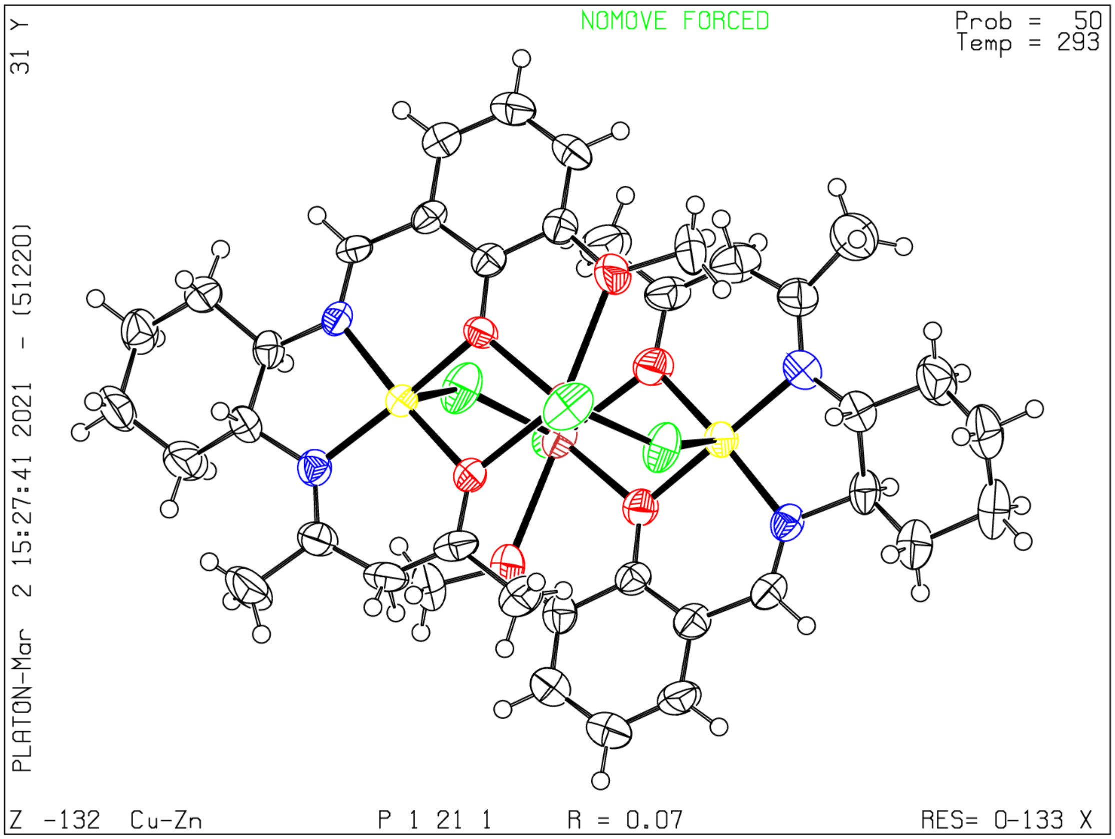

Supplement: Supplementary file 1 [file molecules-26-03825-s001.zip › Crystal/checkcif-Zn.pdf]
