# Supplementary material for: A Highly Selective Turn-On Fluorescent Probe for the Detection of Zinc
Source: Molecules. 2021 Jun 23;26(13):3825. doi: 10.3390/molecules26133825 (PMC8270291; doi:10.3390/molecules26133825)
Supplement: Supplementary file 1 [file molecules-26-03825-s001.zip › molecules-1251598-supplementary.pdf]

molecules

Supporting Information

A Highly Selective Turn-On Fluorescent Probe for the Detection of Zinc

Ling-Yi Shen [1,2,†], Xiao-Li Chen [2,†], Xian-Jiong Yang [1,2,*], Hong Xu [2], Ya-Li Huang [2], Xing Zhang [2], Carl Redshaw [3] and Qi-Long Zhang [1,2,*]

[1] School of Public Health, the key Laboratory of Environmental Pollution Monitoring and Disease Control, Ministry of Education, Guizhou Medical University, Guiyang 550014, China; shenly@stumail.nwu.edu.cn

[2] School of Basic Medical Science, Guizhou Medical University, Guiyang 550004, China; C13885297112@163.com (X.C.); xuhong@gmc.edu.cn (H.X.); ylh6401@gmc.edu.cn (Y.-L.H.); zhangxing11207115@126.com (X.Z.)

[3] Department of Chemistry, University of Hull, Cottingham Road, Hull, Yorkshire HU6 7RX, UK; c.redshaw@hull.ac.uk

* Correspondence: yangxianjiong@gmc.edu.cn (X.-J.Y.); sciqlzhang@gmc.edu.cn (Q.-L.Z.); Fax: +86-0851-88174017 (Q.-L.Z.)

† These authors contributed equally to this work.

Contents:

**^{1}H-NMR spectrum of probe L**	**S1**
**^{13}C-NMR spectrum of probe L**	**S2**
**HR ESI-MS spectrum of probe L**	**S3**
**Absorbance of L as a function of [Zn^{2+}]/[L].**	**S4**
**Job's plot of probe L with Zn^{2+}.**	**S5**
**^{1}H-NMR spectra of probe L with 0–1 equiv. of $ZnCl_2$**	**S6**
**Crystallographic data and refinement details.**	**Table S1**
**Selected bond lengths and bond angles for complex L, Cu-complex and Cu-Zn complex**	**Table S2**

NMR spectrum

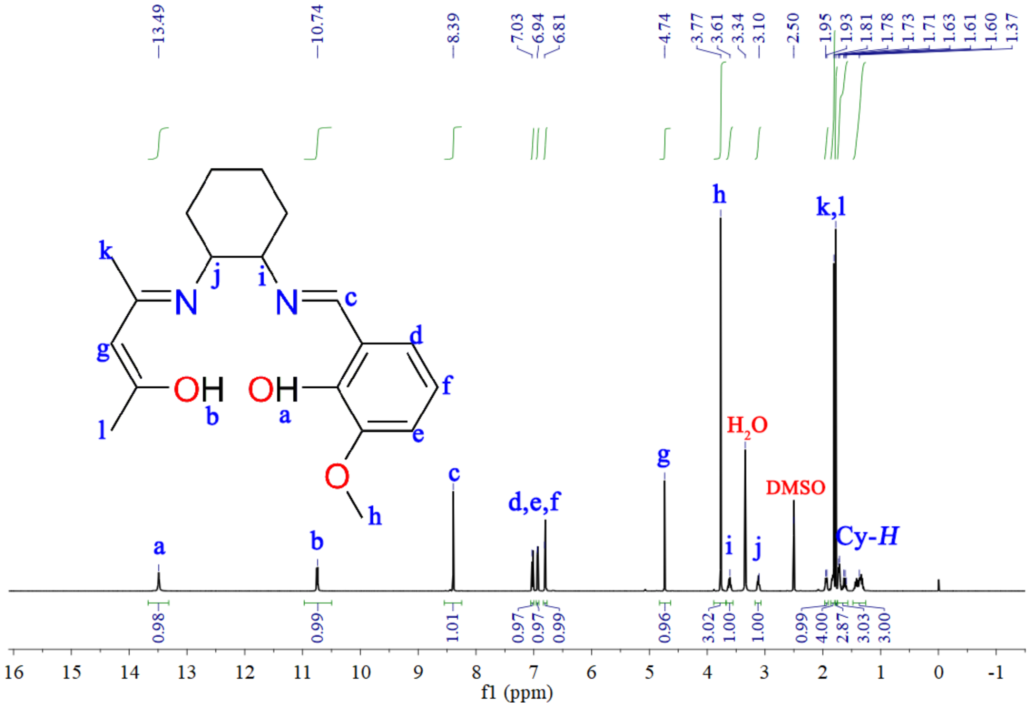

Figure S1. ^{1}H-NMR spectrum of probe **L** (600 MHz, DMSO-d_6, 298 K).

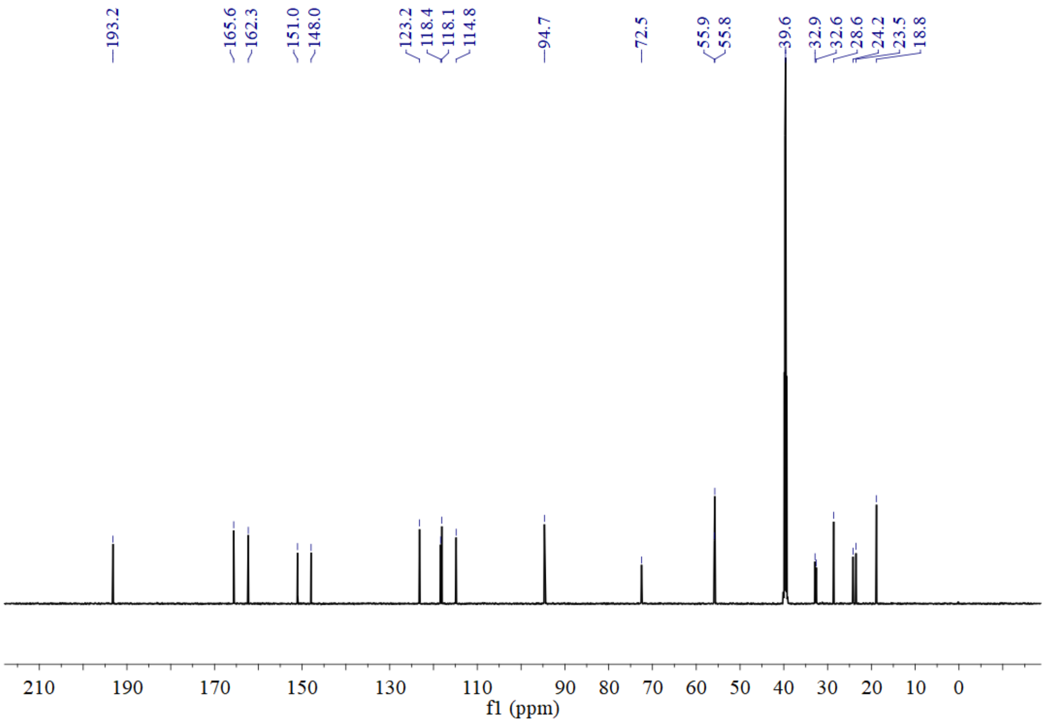

Figure S2. ^{13}C-NMR spectrum of probe **L** in (150.9 MHz, DMSO-d_6, 298 K).

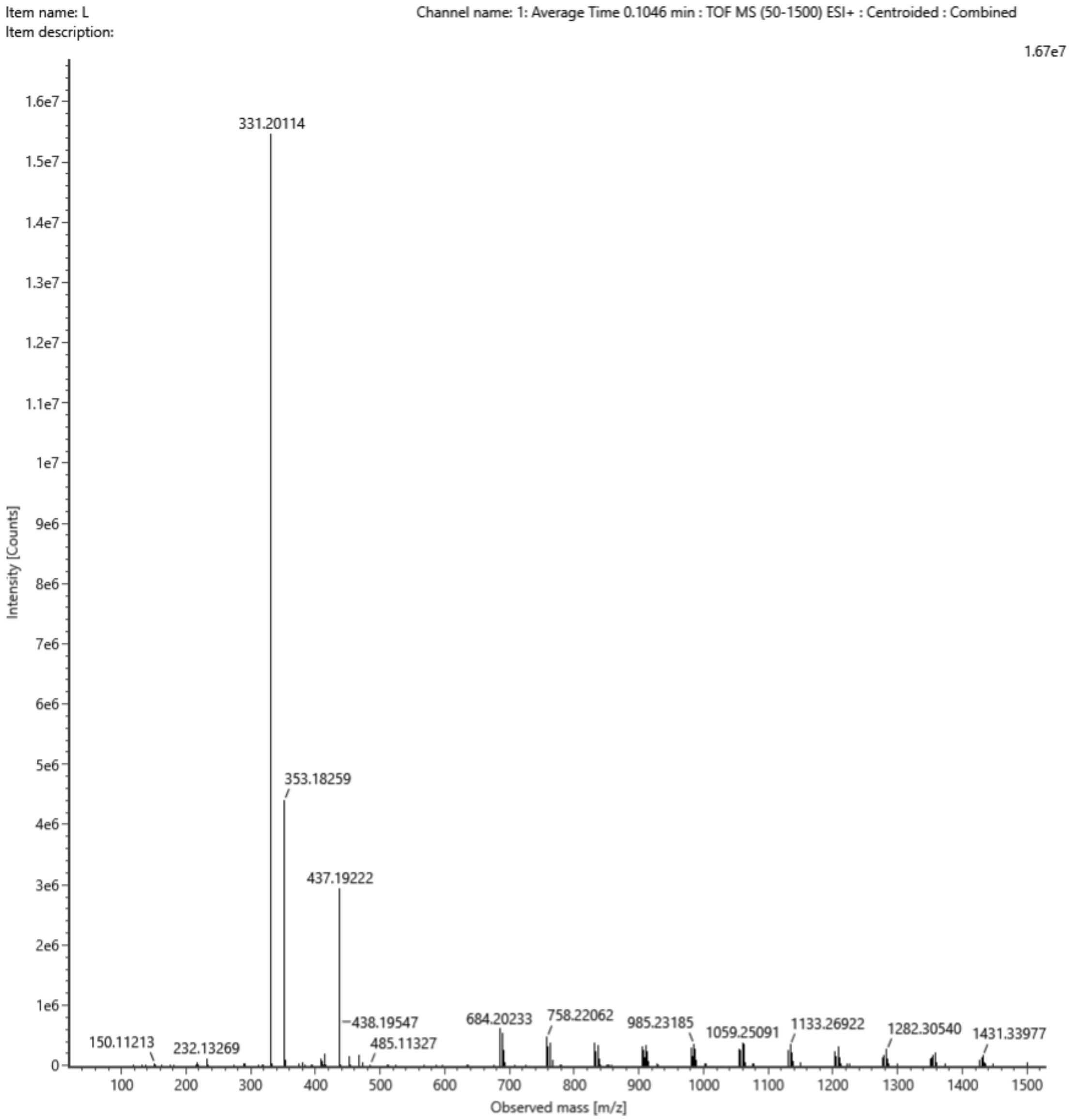

Figure S3. HR ESI-MS spectrum of probe **L.** The *m*/*z* peaks at 331.2011 and 353.1826 correspond to $[M + H]^+$ and $[M + Na]^+$.

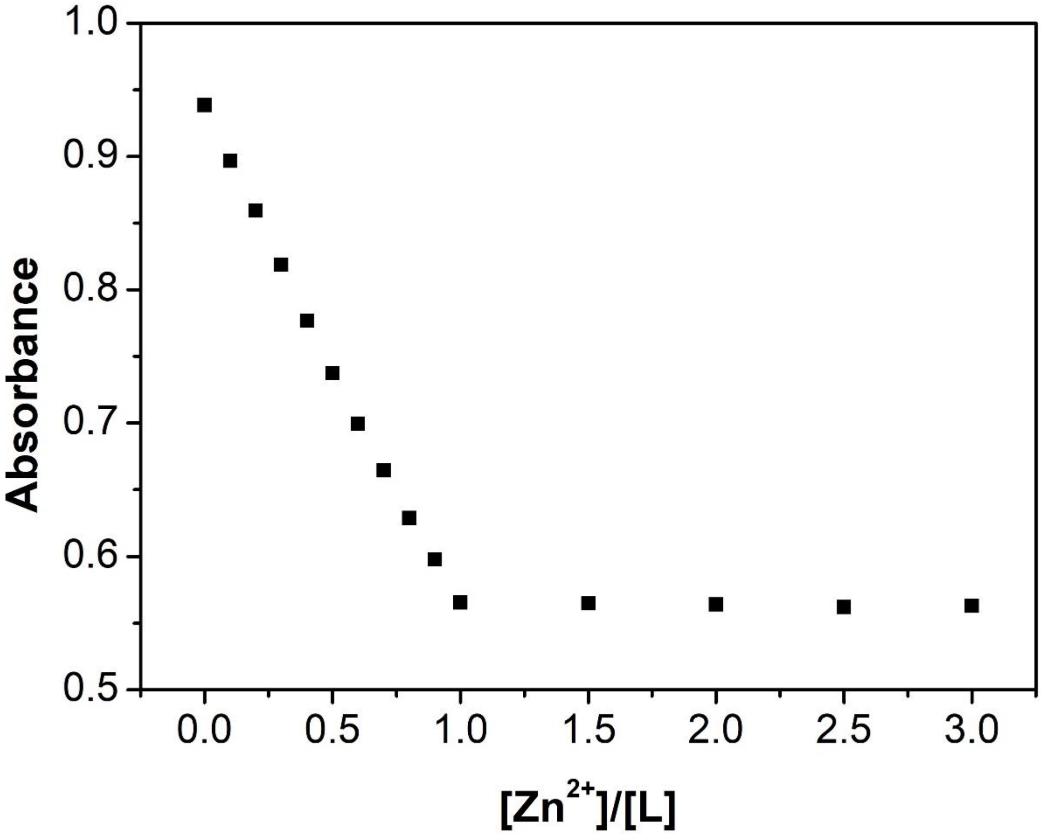

Figure S4. Absorbance of **L** at 312 nm as a function of $[Zn^{2+}]/[L]$.

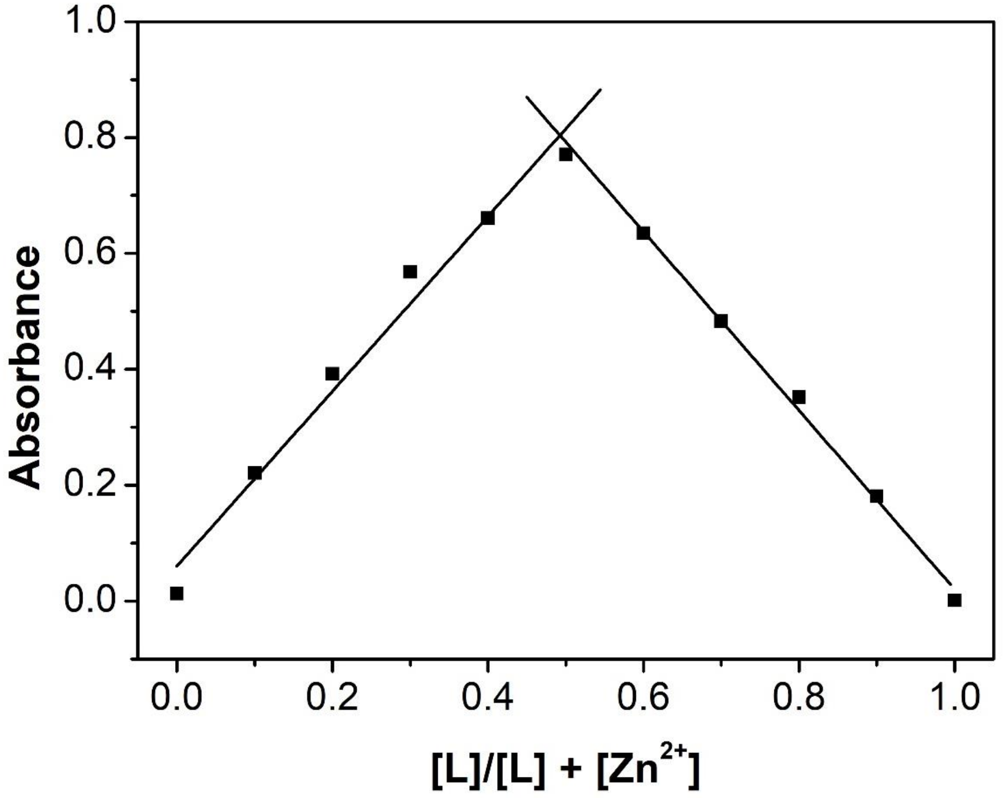

Figure S5. Job's plot of probe **L** with Zn^{2+}.

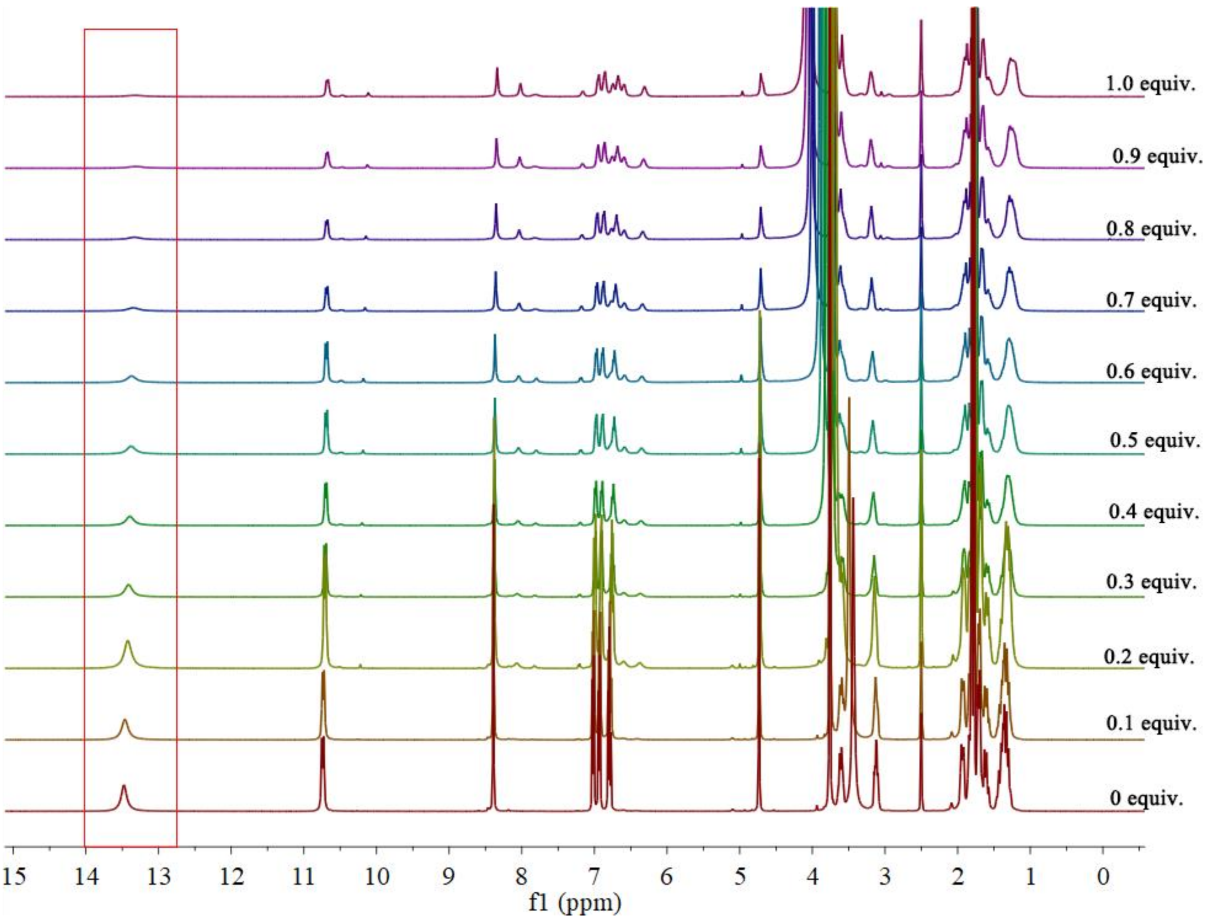

Figure S6. ^{1}H-NMR spectra of probe **L** with 0–1 equiv. of $ZnCl_2$ in DMSO-d_6.

X-ray crystallography Analysis

Table S1. Crystallographic data and refinement details for **L**, Cu-complex and Cu-Zn complex.

Complex	L	Cu-complex	Cu-Zn complex
CCDC Number	2067620	2067621	2067622
Formula	$C_{19}H_{27}N_2O_{3.50}$	$C_{19}H_{24}CuN_3O_3$	$C_{38}H_{50}Cl_4Cu_2N_4O_6Zn_2$
Formula weight	339.42	405.95	1058.44
Crystal system, space group	Tetragonal $P\ 4_2\ 2_1\ 2$	Orthorhombic $P\ 2_1\ 2_1\ 2_1$	Monoclinic $P2_1$
a /Å	17.989(2)	7.6261(8)	9.9812(11)
b /Å	17.989(2)	12.8842(15)	19.966(2)
c /Å	12.862(2)	19.144(2)	11.0609(12)
α /deg	90	90	90
β /deg	90	90	105.840(4)
γ /deg	90	90	90
V /Å^3	4162.2(11)	1881.1(4)	2120.6(4)
Z	8	4	2
D_{calcd} /g cm^{-3}	1.083	1.433	1.658
F(000)	1464	848	1080
μ /mm^{-1}	0.075	1.184	2.408
θ range	2.264–24.992	2.875–28.348	1.914–24.996
R_{int}	0.1916	0.0351	0.1772
Final R_1, wR_2 values ($I > 2\sigma(I)$)	0.1018, 0.2793	0.0382, 0.0668	0.0718, 0.1716
R_1, wR_2 (all data)	0.2233, 0.3231	0.0657, 0.0761	0.0830, 0.1799
GOF (F^2)	1.024	1.029	0.999

Table S2. Selected bond lengths(nm) and bond angles (°) for complex **L**, Cu-complex and Cu-Zn complex.

		**L**			
C1−N1	1.489(10)	C6−N2	1.466(11)	C15−C16	1.348(15)
N1−C17	1.319(10)	N2−C7	1.310(12)	C15−C16	1.348(15)
C15−O1	1.293(14)				
C1−N1−C17	127.0(7)	C6−N2−C7	120.2(8)	N1−C17−C16	121.5(9)
N2−C7−C8	121.9(10)				
		**Cu-complex**			
C1−N1	1.480(5)	C6−N2	1.460(5)	C15−C16	1.352(6)
N1−C17	1.309(5)	N2−C7	1.290(5)	N1−Cu1	1.959(3)
N2−Cu1	1.916(3)	O1−Cu1	1.913(3)	O2−Cu1	1.909(3)
C15−O1	1.292(5)				
C1−N1−C17	123.2(3)	C6−N2−C7	123.4(3)	N1−Cu1−O1	95.25(13)
C1−N1−Cu1	112.6(2)	C6−N2−Cu1	110.1(2)	N1−Cu1−N2	85.22(13)
N2−Cu1−O2	92.44(12)	O1−Cu1−O2	87.78(11)		
		**Cu-Zn complex**			
C1−N1	1.472(11)	C6−N2	1.479(10)	C15−C16	1.372(15)
N1−C17	1.297(12)	N2−C7	1.258(10)	N1−Cu1	1.946(7)
N2−Cu1	1.923(7)	C15−O1	1.267(11)	O1−Cu1	1.917(6)
O2−Cu1	1.934(6)	O1−Zn1	2.146(6)	O2−Zn1	2.002(6)
O3−Zn1	2.441(7)	Cl1−Zn1	2.223(3)	Cl2−Zn1	2.219(3)
Cu1······Zn1	3.1062(13)				

N1−Cu1−O1	97.4(3)	N2−Cu2−O2	93.4(3)	N1−Cu1−N2	87.2(3)
O1−Cu1−O2	81.5(2)	O1−Zn−O2	74.5(2)	Cl1−Zn−Cl2	116.96(11)
